# Development and evaluation of Chitosan nanoparticles based dry powder inhalation formulations of Prothionamide

**DOI:** 10.1371/journal.pone.0190976

**Published:** 2018-01-25

**Authors:** Sujit Kumar Debnath, Srinivasan Saisivam, Monalisha Debanth, Abdelwahab Omri

**Affiliations:** 1 Department of Pharmacy, Bengal College of Pharmaceutical Sciences and Research, Durgapur, West Bengal, India; 2 Department of Pharmacy, N. R. Vekaria Institute of Pharmacy, Junagadh, Gujarat, India; 3 Department of Chemistry & Biochemistry, Laurentian University, Sudbury, ON, Canada; Universidade Estadual Paulista Julio de Mesquita Filho, BRAZIL

## Abstract

Prothionamide (PTH), a second line antitubercular drug is used to administer in conventional oral route. However, its unpredictable absorption and frequent administration limit its use. An alternate approach was thought of administering PTH through pulmonary route in a form of nanoparticles, which can sustain the release for several hours in lungs. Chitosan, a bio-degradable polymer was used to coat PTH and further freeze dried to prepare dry powder inhaler (DPI) with aerodynamic particle size of 1.76μm. *In vitro* release study showed initial burst release followed by sustained release up to 96.91% in 24h. *In vitro* release further correlated with *in vivo* study. Prepared DPI maintained the PTH concentration above MIC for more than 12h after single dose administration and increased the PTH residency in the lungs tissue more than 24h. Animal study also revealed the reduction of dose in pulmonary administration, which will improve the management of tuberculosis.

## Introduction

Most infections in humans result in an asymptomatic, latent infection, and about one in ten latent infections eventually progress to active disease [1]. Tuberculosis usually attacks the lungs and cause pulmonary tuberculosis. Tuberculosis is one of the main causes of mortality and morbidity globally. According to World Health Organization (WHO), *M*. *tuberculosis* has infected approximately one-third of the world’s population, effecting more than 9 million new cases and 2 million deaths annually [2]. In the treatment of tuberculosis, first line drugs like Isoniazid, Rifampicin, Ethambutol, Pyrazinamide and Streptomycin are normally used with large and repeated dose for several months. Due to patient’s non-compliance, these drugs fail and results in drug resistance. In that situation, 2^nd^ line anti-tubercular drugs are generally prescribed. Prothionamide (PTH) belongs to this category, has good clinical efficacy against *Mycobacterium tuberculosis*. It has been used for more than 30 years in the treatment of patients with drug-resistant TB [3]. Due to low biological half life of 2 hr, PTH used to administer repeatedly and this drug remain free form in the systemic circulation that rise the systemic toxicity. The availability of PTH in lungs is very low [4] when given by conventional oral route. An alternative approach was thought to change the route (pulmonary) and dosage form (nanoparticles) for effective management of tuberculosis to make the project a novel one. Nanoparticulate form of PTH can enter intracellular compartments and escape phagocytic macrophages. Previously, Poly lactic co-glycolic acid (PLGA), Resomer RG-755S was tried to coat the PTH. But, this PLGA grade contains 73–77% D, L Lactide and 23–27% Glycolide. Due to lesser glycolide units, it took more time for degradation [5]. As a result, 43.52% PTH released from PLGA nanoparticles in 24h as reported previously [6]. So there was a chance of dose dumping in daily basis intake. To overcome this problem, an alternate bio-degradable polymer was needed, which could release 90–100% PTH in 24h. Chitosan, a partially deacetylated derivative of chitin composed of N-acetylglucosamine, has emerged as a significant biomaterials and pharmaceutical excipient for drug delivery because of its biocompatibility, low immunogenicity and low cost [7]. Chitosan has abundant applications in the field of pharmaceutical and medicine. It is used as a film coating material, as an excipient in tablet, like disintegrant, improvement of drug dissolution, and for controlling drug release [8]. It also been used for capsule, microsphere/ microparticles, nanoparticles, beads, films and gel [9]. Chitosan successfully use in different organ targeted delivery like liver, kidney, lung etc [10]. Moreover, it is mucoadhesive and can promote macromolecule permeation through well organized epithelia [11]. This polycationic polymer can interact with negatively charged substances to form a core shell nanostructure which has been proved to be a promising carrier for drugs [12]. Chitosan coated PTH nanoparticles provides higher drug loading, sustained release, enhanced drug stability and well targeted deposition [13].

## Materials and method

### Materials

Prothionamide was purchased from YarrowChem, Mumbai, India. Low molecular weight of Chitosan (>75% deacetylation) and Sodium Tripolyphosphate (TPP) were purchased from Himedia and Sigma Aldrich respectively. Inhalable grade Lactose anhydrous (INH 40 M 55.115) was obtained as gift sample from Kerry group, USA. All other chemicals were used of analytical grade from Merck Millipore, Mumbai, India. Mono-dose inhaler and nasal insufflators were obtained as a gift sample from MIAT S. P. A. Milano, Italy.

### Compatibility study

Fourier transform infrared spectroscopy (FTIR) analysis was performed to check the chemical interaction between Prothionamide, inhalable anhydrous Lactose and Chitosan using Perkin Elmer (Massachusetts, USA). Two samples were run separately for pure Prothionamide and physical mixture of Prothionamide, inhalable anhydrous Lactose and Chitosan. The samples were scanned in the IR range from 400 to 4000 cm^−1^.

### Preparation of Prothionamide nanoparticles

Chitosan/TPP nanoparticles were prepared using ionic gelation technique with minor modification as described previously [13–14]. Briefly, Chitosan (5mg/ml) was dissolved in aqueous solution of Acetic acid under magnetic stirring at room temp. Prothionamide was dispersed in the above Chitosan solution with constant stirring. TPP aqueous solution was added drop wise using syringe needle into the above Chitosan solution containing PTH with continuous stirring. The stirring was continued for specified period of time. The resultant nano-suspension was centrifuged at 31150g (relative Centrifugal Force) for 30 min followed by one time washing with distilled water. Excessive heat is produce during the centrifugation process, hence to stabilize the nanoparticles -4°C was maintained during centrifugation. Freeze dried the prepared suspension using Mannitol 2% w/v as cryprotectant. In this process primary drying was carried out at -20°C and secondary drying was carried out at -60°C. Several process parameters like stirring time, Chitosan-TPP ratio, Chitosan-PTH ratio and TPP solution volumes were optimized to achieve suitable nanoparticles for the formulation of dry powder inhaler.

#### Characterization of Prothionamide nanoparticles

Percentage Drug Entrapment (PDE) of PTH in nanoparticles was determined by separating the nanoparticles from the aqueous medium by ultracentrifugation at 31150g for 30 min. The amount of free PTH in the supernatant was measured by UV-Spectrophotometery at 288nm [15]. Other evaluation parameters were calculated as reported previously [16]. Zeta potential indicates the surface charge on the particles and was measured to determine the stability of nanoparticles in the suspension [17]. PDI value indicates the particle size distribution of nanoparticles in a given sample. Higher value of PDI indicates the distribution of nanoparticles with variable size range which results in the formation of aggregates and could result in low stability of particle suspension and low homogeneity. The zeta size, zeta potential and Poly Dispersibility Index (PDI) were determined using Zetasizer (Malvern, UK) as mentioned earlier [17].

#### Selection of strength of Acetic acid, volume of Acetic acid and TPP solution

Chitosan dissolves completely in acidic media. Different percentage of Acetic acid solution [18,19,20] was reported in the formulation of Chitosan nanoparticles. As Prothionamide and Chitosan both dissolve in Acetic acid, its percentage was selected as 1% v/v. Initially, Acetic acid and TPP solution volume was selected as 10mL and 5mL respectively [21].

#### Effect of stirring time

Stirring time plays a vital role to obtain uniform PTH nanoparticles. So stirring time was changed by keeping all other process parameters like Chitosan-TPP ratio of 6:1, TPP solution volume of 5 mL, Chitosan-PTH weight ratio of 50:50 same. The influence of stirring time on the average particles size, zeta potential and PDE were studied in four stirring time at 15, 30, 60 and 45 min [13,14,22,23,24]. Based on smaller particle size and higher PDE, optimized formulation had been chosen.

#### Effect of Chitosan and TPP ratio

PTH nanoparticles further optimized for Chitosan-TPP ratio. During this optimization other process parameters were remained constant. From literature, [22–25] four new ratios like 5:1, 4:1, 3:1, and 2:1 were selected. Nanoparticles were prepared by using these ratios and compared with 6:1 ratio optimized after stirring time. Acetic acid and TPP solution volume was maintained 10mL and 5mL respectively. Nanoparticles were prepared using this ratio and studied the influence of it on the physiological character of nanoparticles.

#### Effect of PTH: Chitosan ratio

PTH: Chitosan ratio was optimized accordingly, where all other process parameters were remained constant. Four different formulations were prepared by changing the amount (60, 70, 80 and 90 mg) of Chitosan with fixed amount of PTH and also checked the influence of Chitosan amount on PDE and particles size. As the volume of 1% Acetic acid was restricted to 10ml, maximum 90mg Chitosan could incorporate. Hence, the PTH-Chitosan ratio 1:1.2, 1:1.4, 1:1.6 and 1:1.8 were prepared and compared with 1:1 (CT2).

#### Effect of TPP solution volume

Volumes of TPP and Chitosan solution play a great role in the drug entrapment and uniform particle size distribution. So, volume of TPP solution was increased from 5mL to 10mL (with constant TPP amount) to check its effect on the physicochemical properties of PTH nanoparticles.

#### Scanning electron microscopy (SEM)

The shape and surface morphology of the Prothionamide nanoparticles were examined by Scanning Electron Microscopy (Karl Zesis with SE detector, EVO-18). The samples were sputter-coated with gold and observed for morphology at an acceleration voltage of 7.0 kV with highest magnification of 19.99 KX.

### Formulation of dry powder inhaler and characterization

Initially flow property of PTH nanoparticles was determined. To increase the flow property, PTH nanoparticles and anhydrous inhalable grade lactose were mixed (1:0.5, 1:1, 1:1.5 etc.) manually using geometrical dilution process. Angle of repose, Carr’s index and Hausner ratio were carried out in each stage of addition of inhalable grade lactose (**S1 File**) [26]. Optimization of dry powder inhaler was carried out based on the excellent flow property. Optimized formulation further characterized for the zeta size and potential to check the changes of nanoparticle in the form of DPI.

#### Determination of MMAD using cascade impactor

Mass median aerodynamic diameter (MMAD) represents aerodynamic diameter below which 50% particles remain. Aerodynamic diameter of a particle controls its deposition in pulmonary tract. MMAD of the optimized dry powder inhaler was determined using an eight stage cascade impactor [27]. Firstly, DPIs were flowed through the cascade impactor with a flow rate of 28.3 L/min. After deposition of DPI, Prothionamide content was determined in each chamber by high performance liquid chromatography (HPLC) as reported previously with minor modification [22]. The mobile phase selected was water: acetonitrile at 70:30 with a flow rate 1.0ml/min. Absorbance was detected at 290nm. Estimated drug content in each chamber were inserted into the “MMAD CALCULATOR” to obtain MMAD and geometric standard deviation (GSD). Other parameters like fine particle fraction, extra fine particles were determined as per our previously published articles [28]. Drugs present in the all the stage and filter was used to calculate the emitted dose [29].

#### *In vitro* release study

5mg PTH equivalent dry powder inhaler was dispersed in 2ml of simulated lung fluid (SLF) [30] and filled in dialysis bag (Himedia labs, Mumbai, 12,000 molecular weight cut off) afterward. To avoid floating, the drug filled bag was tied over the glass plate and kept at the bottom of the dissolution chamber. *In-vitro* release study was carried out in 1l SLF [28] maintained at 37±0.5°C with a paddle speed of 100 rpm [31]. 10 ml of dissolution fluid was withdrawn at specific time interval up to 24h and replaced with fresh SLF. The *in-vitro* release data was analyzed for zero order, Highuchi and Korsmeyer-Peppa’s models. Based on the correlation co-efficient (r^2^) best fitted model was selected.

#### Accelerated stability study

Accelerated stability study was carried out as per the ICH guideline Q1A (R2). Paraffin tap was used to seal cryoprotectant vials, contained freshly prepared dry powder inhaler of Prothionamide nanoparticles. These vials were kept in stability chamber and maintained at 25*±*2°C, 60*±*5% RH. The nanoparticles were analyzed for 6 months with a frequency of 1.5 months for first 3 months and next on 6^th^ month [31–33]. Zeta size, zeta potential, PDI, drug entrapment and drug release were carried out to check the stability of dry powder inhaler of Prothionamide nanoparticles.

### *In vivo* study

#### Animals

All animal experiments were approved and performed in accordance with the guidelines of institutional animal ethical committee of Bengal College of Pharmaceutical Sciences and Research, Durgapur, West Bengal (Registration No:1799/PO/Ere/15/S/CPCSEA under CPCSEA, India). Wistar rats either sex were used to study the pharmacokinetic parameters of Prothionamide in the form of DPI. (4–6 months old & average weight 200-250gm). The rats were housed in a 12-h light/dark cycle with food and water available.

#### Pharmacokinetic study

Human lungs volume 4.341 l [34] was used to calculate the human dose of Prothionamide for pulmonary administration. Targeted concentration was chosen as 2μg/ml to maintain the plasma concentration above minimum inhibitory concentration (MIC) for the period of 24h. Dose was divided as loading and maintenance dose. Equivalent dose calculation for rat had been carried out from the human dose. Dose calculation for rat was carried out with equivalent surface and weight with no observed adverse effect with 7.7mg/kg body weight [34–36]. Mono-dose inhaler and nasal insufflators were obtained to administer drug to pulmonary route, but this device was suitable for the human use not for animal administration. So, dry powder inhaler delivery device was designed with little modified as described previously [37]. Rats were divided into two groups [38] one would treated with DPI of Prothionamide nanoparticles and another with pure Prothionamide. Animals were anesthetized intra-peritorial administration of Ketamine (80mg/Kg) and Xylazine (10mg/kg) (**S2 File** & **S3 File**). Gently pulled the tongue outside and sprayed DPIs through the device by placing it in trachea region. In each time period, three animals were selected to obtained statistical significant data. Three separate studies were performed to check the bio and tissue distribution of PTH. Firstly 1ml of blood was withdrawn from the tail vain of rat to check the bio-distribution of PTH at the time interval of 1, 2, 3, 6, 12 & 24h. After sacrifice in this specified interval, lungs were isolated from rat and filled it with 4ml phosphate buffer solution (PBS) by injected slowly in fractions to fill the lungs [39]. The fluid was withdrawn very slowly using syringe and needle and followed by one repetition. This fluid was stirred for 20 min in presence of 1ml methanol. Thereafter, these lungs and trachea were homogenized with 10 ml of PBS solution and 1ml methanol. 60μl of 30% Trichloroacetic acid was added in each sample for deprotenization. After vortexed for 5 min, these samples were centrifuged at 6153g for 10min. With the supernatant, 1 molar sodium bi-carbonate was added to neutralize the sample and followed by vortexed for 5 min. These samples were centrifuge again at 6153g for 10 min. Supernatant containing Prothionamide was estimated using HPLC.

### Statistical analysis

The data were expressed as the mean of three experiments ± standard deviation (S.D.) and were analysis by one-way analysis of variance (ANOVA) for repeated measurements and differences were considered to be significant at a level of P < 0.05.

## Results and discussion

### Compatibility study

**Fig 1:**
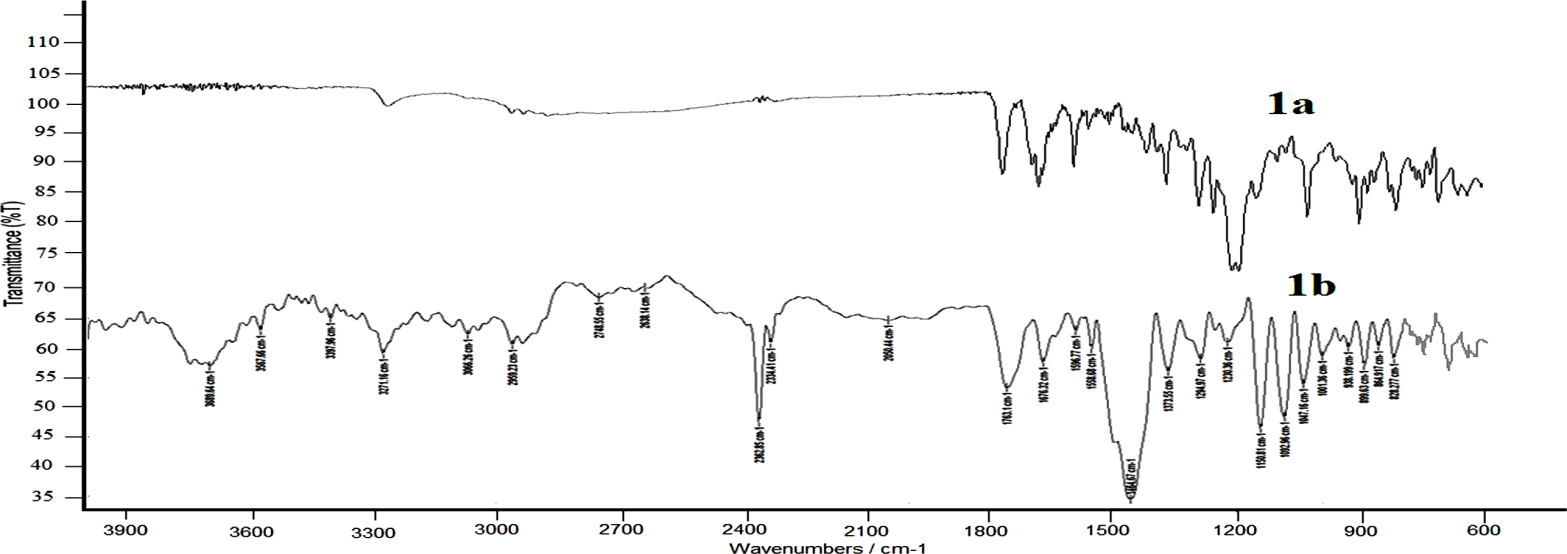
FTIR of pure PTH (1a) and physical mixture of PTH, Chitosan, Inhalable grade lactose (1b).

FTIR spectra (**Fig 1A & 1B**) confirmed the absence of chemical interaction whereby confirming the chemical compatibility by maintaining the integrity of principle peaks. N-H wag peak found in pure PTH was at 823.46 cm^-1^, whereas this was at 828.277 cm^-1^ in physical mixture. = C-H bending was in pure PTH and mixture was 900.59 cm^-1^ & 899.63 cm^-1^ respectively. C-C = C stretch in ring, N-H stretch and C = S stretching peak also showed at 1592.91 cm^-1^, 3266.82 cm^-1^and 1024.02 cm^-1^ respectively with narrow diffraction range which signified the compatibility of PTH with Chitosan and Lactose.

### Characterization of Chitosan nanoparticles

Chitosan nanoparticles were prepared by gelation technique. Cross linking occurred instantly upon addition of Chitosan-PTH solution into the TPP solution. Several step by step optimizations applied to prepare the suitable nanoparticles for dry powder inhaler.

#### Effect of stirring time

There was an increase in PDE initially and that decreased thereafter with increasing the stirring time, while average particle sizes were decreased. Dynamic light scattering (DLS) results are often expressed in terms of the z-average, which is intensity based harmonic mean. The PDI or polydispersity index is an indication of how narrow a Gaussian size distribution would be that could represent the fitted DLS data. There was a significant gap between z-average value and average particle size due to high PDI (**S1 Table**). Formulation C2 showed highest PDE of 63.5 ± 0.56% in comparison to other formulation and its zeta potential value was 30.22 ± 1.88 mV. Most of the cases, values are not statistical significant. Values depicted in the **S1 Table** with *p* value. So, further modification was carried out on C2 formulation.

#### Effect of Chitosan-TPP ratio

Keeping the stirring time of 30 min (C2), four new batches were prepared using different Chitosan/TPP ratio and compared with the formulation C2 (**S2 Table**). There was no significant difference between the C2 and CT2 based on the average particle size but the PDI of CT2 is 0.671 ± 0.006. Hence CT2 had narrow distribution in compare with C2 and resulted in z-average value of 1065.67 ± 41.1nm. Initially average particle size was decreased but this value again increased with increasing the TPP amount. On the other hand, Zeta potential value was decreased due to increase of negative charge TPP amount. In CT3 average particles size was 236.57 ± 28.97nm, but due to PDI value significant correlation with z-average value. Its zeta potential reached -9.41 ± 2.14mV, which had more tendencies to coagulate in solution form rather than CT2. Due to these reason further modification was done on CT 2 batch. Statistical significant found in terms of PDI and in some values of percentage drug entrapment.

#### Effect of PTH-Chitosan ratio

PTH amount was constant in all the formulation, whereas Chitosan amount was increased gradually up to 90 mg in 10mL 1% Acetic acid solution. PTH entrapment was increased significantly with increasing of Chitosan amount. No significant correlation was established between average particle size and z-average in DC6-DC9 due to high PDI. Average particle size and drug entrapment and zeta potential were under consideration for optimization. DC 6 contained least average particles size but PDE was less in compression to DC 8. Hence, DC 8 (contain 80mg of Chitosan) was selected as optimized batch due to smaller particle size and highest PDE (**S3 Table**). In all the case the PDI was near to 1.000, results correlation between average particle size and z-average value was not established. Some of the vales were statistical significant as *p* values <0.05 mentioned in **S3 Table**.

#### Optimization of TPP solution volume

Significant changes occurred in z-average value and PDI with increasing the volume of TPP solution up to 10mL (TPP amount constant). This was the final step for optimization of PTH nanoparticles. Optimized batch (OP) contains 50mg of PTH, 80mg of Chitosan and 16 mg of TPP in 10mL of aqueous solution. Stirring time was maintained 30min. OP batch showed z-average value of 314.37 ± 3.68nm which was significantly correlated with average particle size of 303.77 ± 7.38 nm. PDE, Zeta potential of the optimized batch was found to be 80.65 ± 0.4% and 32.4 ± 1.04mV respectively. The result of drug entrapment, PDI and z- average values of OP batch were found to be statistical significant. Optimized nanoparticles had lesser affinity to coagulate during storage and solution.

#### Scanning electron microscopy (SEM)

**Fig 2:**
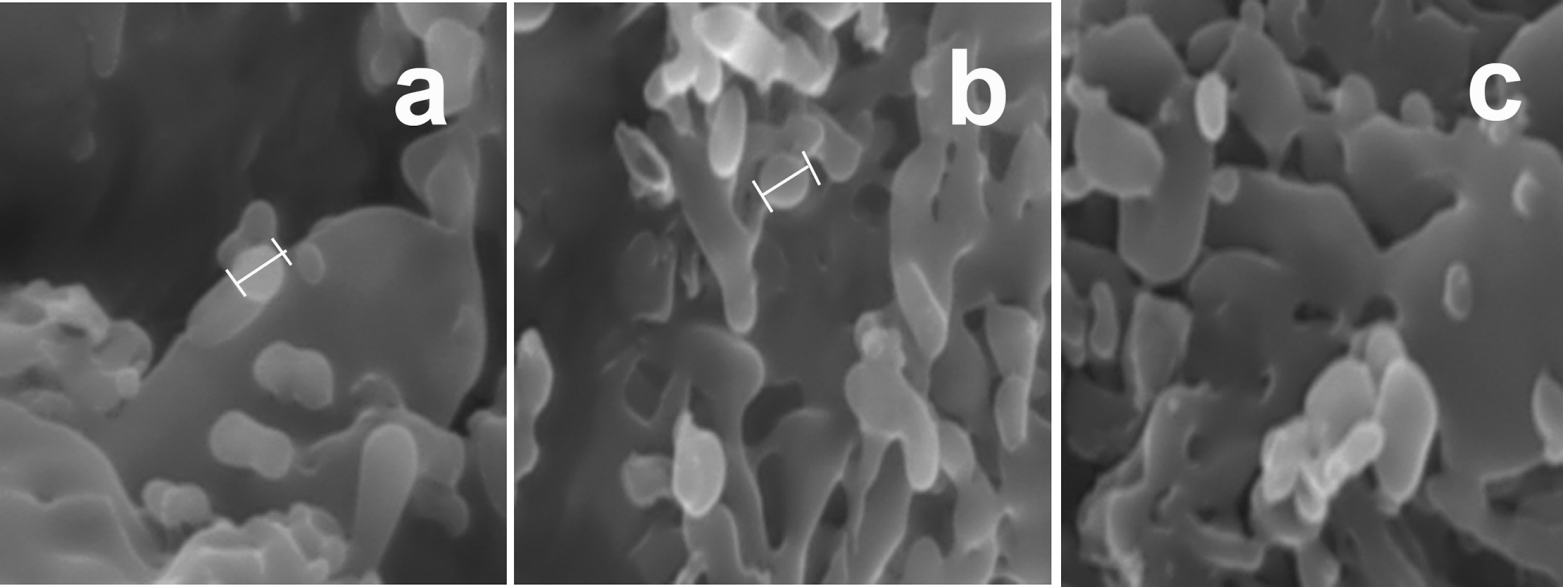
(a & b) SEM image of Chitosan nanoparticles of PTH; (c) SEM image after 6 months.

SEM images of optimized formulation (OP) showed that the nanoparticles were spherical in shape (**Fig 2**). Most of the particle size was of 301.9 nm, which are suitable for the inhalation formulation. Rest of the cases aggregation was occurred due to moisture entrapment during handling.

### Formulation and characterization of dry powder inhaler

The development of an adequate carrier system has become a prerequisite for deep lung delivery, and its choice for pulmonary delivery of NPs is still, however, challenging. Apart from the necessary safety regarding contact with lung tissue, the carrier should provide drug stability, ease of handling during filling and processing, adequate aerodynamic properties for proper lung deposition, as well as improved powder flowability which aids in drug dispersion from the inhaler device [13]. Prepared freeze dried nanoparticles were the fluffy mass with fair flow property. These nanoparticles showed angle of repose, Carr’s index and Hausner ratio as 37.32°, 17.43% and 1.21 respectively. Hence, special inhalable grade lactose was used to increase the bulk and flow property of nanoparticles. Due to fine particles of this special grade lactose, flow property was increase significantly. Initially the Different proportion of anhydrous lactose was added to the optimized Prothionamide nanoparticles (OP) by physical geometric mixture. In each addition of anhydrous lactose, angle of repose, Carr’s index and Hausner ratio were determined (S1 File). In the proportion of 1:1 ratio of lactose- PTH nanoparticles these values were found as 29.25±2.15°, 09.76±2.1% and 1.11±0.01 respectively. Zeta analysis was carried out for the prepared DPI containing Prothionamide nanoparticles and was confirmed no significance changes in zeta potential and z-average value.

### Determination of MMAD and geometric standard deviation

Mass median aerodynamic diameter (MMAD) particle size is the main parameters that determine their deposition in the different parts of lungs. Particle size with 0.5–5 μm can reach to the small airways and alveoli [40]. Deposition of DPI was observed in each stage of the chamber. Higher concentration of carrier, the better was the dispersion [41]. Maximum fine particle fraction, extra fine particle fraction and emitted dose were found as 81.19%, 18.91% and 82.37% respectively. Maximum deposition of DPI was seen in stage 5 followed by 4 and 6, hence aerodynamic particle size obtained 1.76 μm with geometric standard deviation of 1.96. A relatively low MMAD coupled with a low GSD is indicative of a tight size distribution centered on a fine particle size, a potentially beneficial combination for efficient delivery [42]. That signifies that the prepared DPI can reach deeply to lungs.

#### *In vitro* release study

Prepared DPI loaded with Prothionamide nanoparticles was subjected for *in-vitro* drug release behavior. Simulated lung fluid of pH 7.4 was used as dissolution medium for evaluation of release pattern of Prothionamide from Chitosan nanoparticles [30]. Prepared Prothionamide-Chitosan nanoparticles showed initial burst release of 22% and released up to 96.91% in 24h. This initial burst release would help to reach the desired plasma concentration in lungs, and the sustained release would help them to maintain the dose for prolong period of time. The model that best fits the release data is selected based on the correlation coefficient value of various models. Initially significant difference observed but latter most of the cases *p* values observed significant. The *in vitro* release data was analyzed through zero order, Highuchi, Korsmeyer-Peppa’s models and obtained correlation co-efficient (*r*^*2*^) were found to be 0.886, 0.961, and 0.976 respectively. The release best fitted Korsmeyer -Peppa’s model based on *r*^*2*^ and release exponent “n” of Korsmeyer-Peppas kinetic model describes drug release mechanism. Values of “n” was 0.544 signifies the release refers to both combination of erosion and diffusion mechanism.

#### Accelerated stability study

Stability of NPs was determined in terms of z-average particle size, zeta potential, drug entrapment and release profile. Zeta size, zeta potential, PDI, and drug release demonstrated the conservation of dry powder inhaler during stress testing. There was no significant changes occurred in PDI, Zeta potential, PDE and % drug release. Particle sizes were changed in narrow range but it did not create any significant effect on the release of PTH from Chitosan nanoparticles (**S4 Table**). 6m accelerated stability study revealed no clumping or aggregation of particles shown in (**Fig 2C**).

### *In-vivo* study

**Fig 3:**
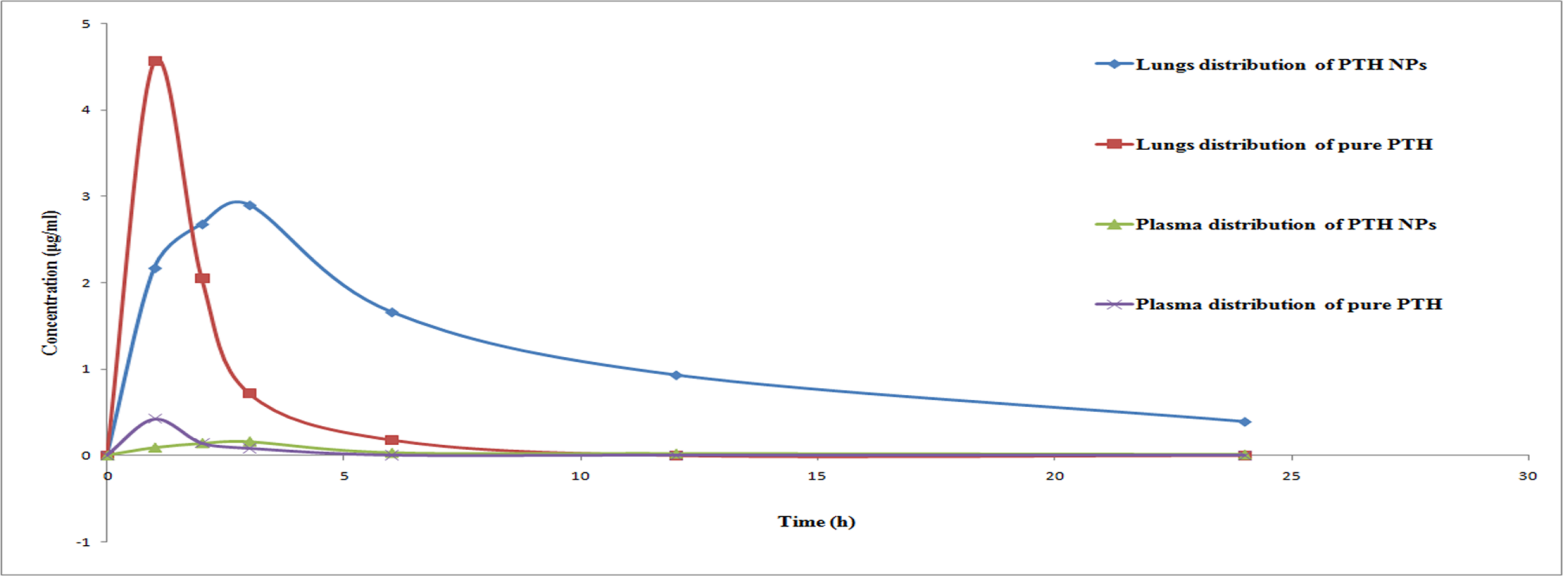
Bio-distribution of PTH in free and nanoparticles form.

Three different studies were carried out to demonstrate percentage drug release *in-vivo*, lungs tissue distribution and bio-distribution of Prothionamide at specific time interval (**S5 Table**). In contrast to pure form, PTH nanoparticles gave sustained release of 97.80 ± 0.51% for the period of 24h, whereas pure PTH gave 99.84±0.08% release in 6h. As a result PTH concentration reached 4.56±0.31 μg/ml (C_max_) at T_max_ 1h in lungs. AUC increased significantly when PTH was given in the form of nanoparticles. There was no significant difference between the AUC of PTH nanoparticles and pure PTH administration by blood samples analysis. This was happened due to rapid release of PTH in the pure form. As PTH reached very slowly to plasma from PTH nanoparticles, AUC achieved relatively less in compression to lungs tissue reviled from (**Fig 3**). Prothionamide nanoparticles loaded DPI achieved maximum concentration 2.90±0.28 μg/ml (C_max_) at T_max_ 3h. DPI of PTH nanoparticles maintained plasma concentration above the MIC for the period more than 12h, whereas pure PTH could maintain up to 3h. PTH nanoparticles gave the concentration of 0.39± 0.06 μg/ml at 24 h, which was lower than the MIC. So, prepared formulation should be used once every day to maintain the plasma concentration above MIC for the period of 24h. Due to short half life, PTH is prescriber 250 mg for 2 times a day in oral route. This animal study confirmed PTH maintained concentration above MIC with reduced does. In terms of concentration of PTH in blood, AUC of plasma and some other parameters values found to be statistical significant with the *p* value less than 0.05 depicted in the **S5 Table**.

## Conclusion

Prothionamide nanoparticles were prepared through ionic gelation technique. Prepared Prothionamide nanoparticles were spherical in shape with particle size 314.37 ± 3.68 nm, which also confirmed from the SEM analysis. Optimized Prothionamide nanoparticles further modified to dry powder inhaler with aerodynamic particle size of 1.76μm and signified its suitability in effective delivery for pulmonary administration. *In-vitro* release study signifies the release occurred due to combination of erosion and diffusion mechanism and followed Korsmeyer-Peppas kinetic model. Particle sizes were changed in narrow range during storage time but it did not create any significant effect on the release of PTH from Chitosan nanoparticles. Prepared DPI maintained Prothionamide concentration above MIC for more than 12h after single dose administration and also can improve the effectiveness of the treatment by increasing PTH concentration in the lungs tissue with reduced dose.

## Supporting information

S1 Table Effect of stirring time. (DOC)

S2 Table Effect of Chitosan & TPP ratio. (DOC)

S3 Table Effect of PTH:Chitosan ratio. (DOC)

S4 Table Accelerated stability study of DPI4 loaded with OP2. (DOC)

S5 Table Pharmacokinetic evaluation. (DOC)

S1 File Flow property determination. (XLS)

S2 File Anaesthetic dose administration in rat treated with DPI. (XLSX)

S3 File Anaesthetic dose administration in rat treated with Pure Prothonamide. (XLSX)
