# Supplementary material for: Development and evaluation of Chitosan nanoparticles based dry powder inhalation formulations of Prothionamide
Source: PLoS One. 2018 Jan 25;13(1):e0190976. doi: 10.1371/journal.pone.0190976 (PMC5784924; doi:10.1371/journal.pone.0190976)
Supplement: S1 Table — (DOC) [file pone.0190976.s001.doc]

**S1 Table. Effect of stirring time**

| **Formula code** | **Stirring time (min)** | **z-average value (nm) ^**^** | **Average particle size (nm) ^**^** | **PDI ^**^** | **Zeta potential (mV) ^**^** | **Entrapment efficiency (%) ^**^** |
| --- | --- | --- | --- | --- | --- | --- |
| C1 | 15 | 7921 ± 174 | 553.9 ± 45.2 | 0.995 ± 0.007^#^ | 31.67 ± 2.52 | 52.31 ± 1.85 |
| C2 | 30 | 3238 ± 102 | 456.9 ± 33.04 | 0.963 ± 0.031^#^ | 30.22 ± 1.88 | 63.5 ± 0.56^#^ |
| C3 | 45 | 1687 ± 55 | 161.5 ± 35.4 | 0.993 ± 0.01^#^ | 18.47 ± 3.81 | 46.99 ± 1.8 |
| C4 | 60 | 5545 ± 97 | 235.37 ± 52.18 | 0.991 ± 0.015^#^ | 26.17 ± 1.97 | 55.69 ± 2.16 |
| ^**^Values depicted as mean with standard deviation;  ^#^*p* value less than 0.05 | | | | | | |
