# Supplementary material for: Development and evaluation of Chitosan nanoparticles based dry powder inhalation formulations of Prothionamide
Source: PLoS One. 2018 Jan 25;13(1):e0190976. doi: 10.1371/journal.pone.0190976 (PMC5784924; doi:10.1371/journal.pone.0190976)
Supplement: S2 Table — (DOC) [file pone.0190976.s002.doc]

**S2 Table. Effect of Chitosan & TPP ratio**

| **Formula code** | **Chitosan: TPP**  **(W/W)** | **z-average value (nm) ^**^** | **Average particle size (nm) ^**^** | **PDI ^**^** | **Zeta potential (mV) ^**^** | **Drug entrapment (%) ^**^** |
| --- | --- | --- | --- | --- | --- | --- |
| C2 | 6:1 | 3238 ± 120 | 456.9 ± 33.04 | 0.963 ± 0.031^#^ | 30.22 ± 1.88 | 63.5 ± 0.56^#^ |
| CT 2 | 5:1 | 1065.67 ± 41.1 | 470.93 ± 93.01 | 0.671 ± 0.006^#^ | 17.71 ± 2.93 | 69.42 ± 1.96 |
| CT 3 | 4:1 | 1635.33 ± 53.53 | 236.57 ± 28.97 | 0.703 ± 0.009^#^ | -9.41 ± 2.14 | 66.37 ± 2.53 |
| CT 4 | 3:1 | 5448 ± 125.74 | 428.7 ± 28.97 | 0.697 ± 0.009^#^ | 1.78 ± 1.03 | 42.38 ± 1.31 |
| CT 5 | 2:1 | 2569.3 ± 133.25 | 443.73 ± 31.01 | 0.732 ± 0.01^#^ | 19.49 ± 2.19 | 42.16 ± 0.77^#^ |
| ^**^Values are mean ± standard deviation;  ^#^*p* value less than 0.05 | | | | | | |
