# Supplementary material for: Development and evaluation of Chitosan nanoparticles based dry powder inhalation formulations of Prothionamide
Source: PLoS One. 2018 Jan 25;13(1):e0190976. doi: 10.1371/journal.pone.0190976 (PMC5784924; doi:10.1371/journal.pone.0190976)
Supplement: S3 Table — (DOC) [file pone.0190976.s003.doc]

**S3 Table. Effect of PTH:Chitosan ratio**

| **Formula code** | **PTH: Chitosan (mg)** | **z-average value (nm) ^**^** | **Average particle size (nm) ^**^** | **PDI ^**^** | **Zeta potential (mV) ^**^** | **Drug entrapment (%) ^**^** |
| --- | --- | --- | --- | --- | --- | --- |
| CT 2 | 50:50 | 1069 ± 54 | 462.2 ± 89.2 | 0.666 ± 0.11^#^ | 15.5 ± 5.6 | 69.69 ± 2.32 |
| DC 6 | 50:60 | 2792.33 ± 39.50 | 187.07 ± 23.92 | 0.992 ± 0.12^#^ | 35.9± 0.79 | 70.9 ± 0.45 |
| DC 7 | 50:70 | 2874.33 ± 60.14 | 256.13 ± 26.5 | 0.997 ± 0.004^#^ | 21.6 ± 1.9 | 73.65 ± 0.5 |
| DC 8 | 50:80 | 2978.67 ± 29.91 | 236.3 ± 10.58 | 0.975 ± 0.029^#^ | 31.37 ± 1.96 | 78.8 ± 0.36^#^ |
| DC 9 | 50:90 | 2882 ± 56.22 | 333.5 ± 23.19 | 0.999 ± 0.001^#^ | 27.4 ± 2.11 | 74.78 ± 0.23^#^ |
| ^**^Values are mean ± standard deviation  ^#^*p* value less than 0.05 | | | | | | |
