# Supplementary material for: Development and evaluation of Chitosan nanoparticles based dry powder inhalation formulations of Prothionamide
Source: PLoS One. 2018 Jan 25;13(1):e0190976. doi: 10.1371/journal.pone.0190976 (PMC5784924; doi:10.1371/journal.pone.0190976)
Supplement: S4 Table — (DOC) [file pone.0190976.s004.doc]

**S4 Table. Accelerated stability study of DPI4 loaded with OP2**

| **Time point (month)** | **z-average (nm) ^**^** | **PDI^**^** | **Zeta potential^**^** | **% Drug Entrapment^**^** | **% drug release in 24 hr^**^** |
| --- | --- | --- | --- | --- | --- |
| 0 | 323.3 ± 10 | 0.411 ± 0.13 | 28.3 ± 3.6 | 79.75 ± 0.11 | 96.91 ± 1.5 |
| 1.5 | 326.7 ± 09 | 0.429 ± 0.21 | 28.6 ± 2.7 | 79.50 ± 0.09 | 98.86 ± 0.9 |
| 3 | 351.3 ± 05 | 0.426 ± 0.32 | 28.4 ± 3.5 | 78.33 ± 0.12 | 97.71 ± 3.2 |
| 6 | 361.4 ± 12 | 0.428 ± 0.47 | 29.5 ± 2.1 | 78.12 ± 0.16 | 98.32 ± 6.4 |
| ^**^Values are mean ± standard deviation with three repetitions | | | | | |
