# Supplementary material for: Development and evaluation of Chitosan nanoparticles based dry powder inhalation formulations of Prothionamide
Source: PLoS One. 2018 Jan 25;13(1):e0190976. doi: 10.1371/journal.pone.0190976 (PMC5784924; doi:10.1371/journal.pone.0190976)
Supplement: S5 Table — (DOC) [file pone.0190976.s005.doc]

**S5 Table. Pharmacokinetic evaluation**

| **Time (hr)** | **Percentage drug release**^**^ | | **Concentration of PTH in lungs (µg/ml)** ^**^ | | **AUC of Pulmonary pharmacokinetic (µg*hr/ml)** ^**^ | | **Concentration of PTH in blood (µg/ml)** ^**;#^ | | **AUC of plasma pharmacokinetic (µg*hr/ml)** ^**;#^ | |
| --- | --- | --- | --- | --- | --- | --- | --- | --- | --- | --- |
|  | PTH nano. | Pure PTH | PTH nano. | Pure PTH | PTH nano. | Pure PTH | PTH nano. | Pure PTH | PTH nano. | Pure PTH |
| 1 | 14.68 ± 1.22 | 38.51 ± 0.59 | 2.17 ± 0.27 | 4.56 ± 0.31 | 29.12 ± 1.96 | 10.08 ± 2.31 | 0.09 ± 0.002 | 0.42 ± 0.03 | 0.89 ± 0.085 | 0.728 ± 0.0089 |
| 2 | 23.57 ± 1.13 | 67.62 ± 2.95 | 2.68 ±0.23 | 2.05 ± 0.09^#^ |  |  | 0.14 ± 0.014 | 0.14 ± 0.01 |  |  |
| 3 | 30.54 ± 2.42 | 91.41 ± 1.22 | 2.90 ± 0.28 | 0.72 ± 0.06^#^ |  |  | 0.16 ± 0.009 | 0.08 ± 0.01 |  |  |
| 6 | 50.40 ± 2.23 | 99.84 ± 0.08^#^ | 1.66 ± 0.09^#^ | 0.18 ± 0.03^#^ |  |  | 0.03 ± 0.001 | 0.001 ± 0.001 |  |  |
| 12 | 72.65 ± 1.48 | --- | 0.93 ± 0.13 | **--** |  |  | 0.02 ± 0.007 | **---** |  |  |
| 24 | 97.80 ± 0.51 | --- | 0.39 ± 0.06^#^ | **---** |  |  | 0.01 ± 0.002 | **---** |  |  |
| ^**^ Values are mean ± standard deviation.  ^#^*p* value less than 0.05. | | | | | | | | | | |
